# Development and Characterization of a Genetically Stable Infectious Clone for a Genotype I Isolate of Dengue Virus Serotype 1

**DOI:** 10.3390/v14092073

**Published:** 2022-09-18

**Authors:** Mingyue Hu, Tiantian Wu, Yang Yang, Tongling Chen, Jiawei Hao, Youchuan Wei, Tingrong Luo, De Wu, Yi-Ping Li

**Affiliations:** 1 College of Animal Sciences and Veterinary Medicine, Guangxi University, Nanning 530004, China; 2 Institute of Human Virology, Department of Pathogen Biology and Biosecurity, Key Laboratory of Tropical Disease Control of Ministry of Education, Zhongshan School of Medicine, Sun Yat-sen University, Guangzhou 510080, China; 3 Institute of Pathogenic Microbiology, Center for Disease Control and Prevention of Guangdong, Guangzhou 511430, China; 4 Department of Infectious Diseases, The Third Affiliated Hospital of Sun Yat-sen University, Guangzhou 510630, China

**Keywords:** dengue virus, reverse genetics, infectious clone, amino acid substitution, genetically stable

## Abstract

Dengue virus (DENV) is primarily transmitted by the bite of an infected mosquito of *Aedes aegypti* and *Aedes albopictus*, and symptoms caused may range from mild dengue fever to severe dengue hemorrhagic fever and dengue shock syndrome. Reverse genetic system represents a valuable tool for the study of DENV virology, infection, pathogenesis, etc. Here, we generated and characterized an eukaryotic-activated full-length infectious cDNA clone for a DENV serotype 1 (DENV-1) isolate, D19044, collected in 2019. Initially, nearly the full genome was determined by sequencing overlapping RT-PCR products, and was classified to be genotype I DENV-1. D19044 wild-type cDNA clone (D19044_WT) was assembled by four subgenomic fragments, in a specific order, into a low-copy vector downstream the CMV promoter. D19044_WT released the infectious virus at a low level (1.26 × 10^3^ focus forming units per milliliter [FFU/mL]) following plasmid transfection of BHK-21 cells. Further adaptation by consecutive virus passages up to passage 37, and seven amino acid substitutions (7M) were identified from passage-recovered viruses. The addition of 7M (D19044_7M) greatly improved viral titer (7.5 × 10^4^ FFU/mL) in transfected BHK-21 culture, and virus infections in 293T, Huh7.5.1, and C6/36 cells were also efficient. D19044_7M plasmid was genetically stable in transformant bacteria after five transformation-purification cycles, which did not change the capacity of producing infectious virus. Moreover, the D19044_7M virus was inhibited by mycophenolic acid in a dose-dependent manner. In conclusion, we have developed a DNA-launched full-length infectious clone for a genotype I isolate of DENV-1, with genetic stability in transformant bacteria, thus providing a useful tool for the study of DENV-1.

## 1. Introduction

Dengue virus (DENV) belongs to the genus *Flavivirus* of the family *Flaviviridae*. It is the most widespread arbovirus mainly transmitted by *Aedes aegypti* and *Aedes albopictus*. DENV infection can cause a spectrum of diseases varying from mild dengue fever to severe diseases, such as dengue haemorrhagic fever (DHF) and dengue shock syndrome (DSS) [1,2]. With global warming and active commercial and traveling flows, the vector mosquitoes have expanded unexpectedly, increasing the risk of global DENV prevalence. In the past 50 years, the incidence of dengue fever has soared 30 times [3]. The World Health Organization (WHO) reports that DENV infection has been identified in 128 countries, mostly in tropical and subtropical regions [1,4,5]. In 2019, the WHO announced dengue as one of the top ten threats to global health [6].

Four serotypes of DENV (DENV-1-4) have transmission cycles in humans, and serotypes differ from each other by 30–35% amino acid (aa) sequence [7,8]. Antibody-dependent enhancement (ADE) and cross-reactive T cell-mediated cytotoxicity may occur when a secondary infection caused by a heterogenous serotype virus [9] or other flaviviruses [10,11,12,13]. ADE is believed as an important risk factor of DHF and DSS [10]. To date, only one vaccine was licensed for DENV, however the issues of effectiveness and safety remain, and it has not been widely used [14]. Moreover, there is no an antiviral drug available for DENV infection.

DENV-1 has five genotypes, I-V, varying by approximately 3% at amino acid level and 6% at nucleotide level [7,8,15]. The emergence of a highly divergent isolate suggested the existence of genotype VI DENV-1, and it may have circulated in multiple regions in China and other countries [16]. Genotype I is the most prevalent genotype of DENV-1 responsible for several major outbreaks in south China since 2000 [1], however only few DENV-1 and one genotype I infectious clones (DENV1-3_C_0268-96, Thailand, 1996) have been developed previously [17]. Reverse genetic systems representative of different serotypes and prevalent isolates will provide valuable tools for the studies of molecular virology, virus-host interactions, viral pathogenesis, cross-species transmission, vaccine and antiviral drugs. Given the antigenic and genetic diversity of DENV, an infectious cDNA clone of clinical isolate from recent outbreaks will be highly relevant, especially for genotype I of DENV-1. However, some technical challenges exist in the construction of a genome length cDNA clone for DENV and other flaviviruses, such as Zika virus (ZIKV), into a plasmid vector, as the viral cDNA sequence may contain cryptic prokaryotic promoters leading to unexpected transcription and expression of proteins toxic or lethal to the transformant recipient bacteria [18,19,20]. As a consequence, the replication of plasmid becomes unstable in the transformant *E. coli* bacteria, and it remains a challenge to overcome the instability of genomic cDNA in bacteria.

In this study, we constructed and adapted a full-length infectious clone of DENV-1 genotype I isolate, D19044, for efficient virus production in cultured cells. The genetic stability in transformant bacteria and responsiveness to antiviral compound were also proven. D19044 was isolated in 2019 from an endemic in Jieyang city, Guangdong province, China; thus, this clone will be highly relevant for the study of DENV-1 in prevalence and those close-related endemics.

## 2. Materials and Methods

### 2.1. Cells and DENV-Infected Serum

Huh7.5.1 cells were provided by Dr. Francis Chisari (The Scripps Research Institute, La Jolla, CA, USA) and Dr. Jin Zhong (Institut Pasteur of Shanghai, Shanghai, China). C6/36 cells were provided by Dr. Ping Zhang (Sun Yat-sen University, Guangzhou, China). BHK-21, A549, 293T, and Vero (Vero, originally refers to ATCC No. CCL-81) cells were maintained in our laboratory. BHK-21, A549, 293T, Huh7.5.1, and Vero cells were cultured in a high-glucose Dulbecco’s modified Eagle’s medium (DMEM) supplemented with 10% fetal bovine serum (FBS) and 1% penicillin/streptomycin, and incubated at 37 °C with 5% CO_2_. C6/36 cells were maintained in a high-glucose DMEM supplemented with 10% FBS, 1% penicillin/streptomycin, and 1 × Non-Essential Amino Acids Solution (Gibco, CA, USA), and incubated at 28 °C with 5% CO_2_. The cells used for experiments were tested routinely by mycoplasma-specific PCR to ensure free of mycoplasma contamination. The DENV-1-infected serum was collected from a patient (no. D19044) by Guangdong Provincial Center for Disease Prevention and Control, China, for the purpose of disease surveillance and prevention, during an endemic in Jieyang city, Guangdong, China, in 2019.

### 2.2. Determination of Viral Genome Sequence and Construction of Full-Length cDNA Clone

The patient serum (no. D19044) was inoculated to C6/36 cells and the culture supernatant from day 6 was divided into two parts. One part of supernatant was assigned for sequencing analysis and subjected to RNA extraction using MagZol Reagent (Magen, Guangzhou, China). The nearly full-length genomic cDNA was synthesized by reverse transcription using SuperScript III Reverse Transcriptase (Invitrogen, CA, USA) (primers R6260*/R10654*) (Table 1). Four overlapping PCRs covering the ORF and partial 5′UTR and 3′UTR sequences were performed using primers F17/R3347, F2724/R4897, F4755/R8347, F7485/R10544, F-5′UTR/R-5′UTR, and F-3′UTR/R-3′UTR (Table 1). The PCR fragments were sequenced by Sanger sequencing method, and nearly full-length genome (nucleotides [nts] 50 to 10508; GenBank no. OP315653) was determined and confirmed by aligning to DENV-1 sequences from GenBank (MT076934.1, KY057373.1, MG679801.1, KY672944.1, KY926849.1, MF405201.1, KX225491.1, KP406801.1, AF226685.2, AY732483.1, and NC_001477.1).

To assemble full-length D19044 wild-type cDNA clone (D19044_WT), nts 1–49 and 10,509–10,735 were synthesized identical to a DENV-1 isolate, GZ2002 (GenBank accession number, JN205310.1) [21]. Amino acid substitutions were engineered into the D19044_WT by fusion PCR or gene synthesis, such as D19044_4M and D19044_7M (GenBank no. OP315654). Using partial 5′UTR and 3′UTR sequences from isolate GZ2002 avoided performing rapid amplification of cDNA 5′ ends (5′RACE) and 3′RACE to determine the terminus sequences of D19044 genome. Although the secondary and tertiary RNA structures in the 5′UTR and 3′UTR of DENV and other flaviviruses play fundamental roles in viral replication and translation [22], the sequences from GZ2002 (nts 1–49 and 10,509–10,735) were located in the conserved regions of DENV genome [23]; D19044 had no difference with GZ2002 in the 5′UTR nts 50–94 and only differed by 11 nts in the 3′UTR nts 10,274–10,508, which were identified in this study. D19044 shared 91.36% and 96.93% sequence identity with GZ2002 at nucleotide and amino acid levels, respectively. In addition, GZ2002 was isolated also from Guangdong province, China, in 2002 [21]. To construct the full-length cDNA clone, we divided the D19044 genome into four subgenomic fragments: fragment A (nts 1–2805, *Sac*I*-Bsr*GI), fragment B (nts 2724–4897, *Bsr*GI*-Bsr*GI), fragment C (nts 4755–8347, *Bsr*GI*-Cla*I), and fragment D (nts 7485–10735, *Cla*I*-Rsr*II). These four fragments were assembled into pTight vector by in-fusion cloning using a One Step Cloning Kit (Vazyme, Nanjing, China), and surprisingly the cloning succeeded only when fragments were assembled in the order of D, C, A, B. All restriction endonucleases were purchased (New England Biolabs, NEB, MA, USA). The pTight vector was originally modified from pBR322, which contains seven repeated tetracycline-response elements (7 × TRE) located upstream of the minimal cytomegalovirus (CMVmin) promoter sequence, and it was used previously for cloning a DENV-2, DENV-2-pTight (provided by Dr. Andrew Yueh, National Health Research Institutes, Taiwan, China) [19].

### 2.3. Transformation and Purification of Plasmids

Full-length D19044 cDNA clone plasmid was transformed into competent Turbo bacteria (NEB, MA, USA) and plated on solid 2 × YT medium plates containing ampicillin (15 μg/mL) at 28 °C for 24 h. The colonies were selected and cultured in liquid 2 × YT medium with ampicillin (15 μg/mL) at 28 °C for 20 h. The plasmid was extracted by HiPure Plasmid Mini kit (Magen, Guangzhou, China) and confirmed by Sanger sequencing analysis.

### 2.4. Phylogenetic Analysis of D19044 and Other DENV Sequences

The sequence of D19044 virus was generated by Sanger sequencing four overlapping RT-PCR products (above). Twelve DENV-1 genotypes I–V isolates with complete genome sequences available in GenBank and other eight sequences from DENV-2, -3, and -4 (two sequences for each) were selected for phylogenetic analysis. Sequences representative of DENV serotypes 2–4 were selected, especially for those with infectious clones developed. The phylogenetic tree was made based on the ORF nucleotide sequences using the neighbor-joining method in the Molecular Evolutionary Genetics Analysis (MEGA, version 6, Kimura 2-parameter). The Basic Local Alignment Search Tool (BLAST, version +2.13.0) was used to analyze the sequences homology to D19044.

### 2.5. Virus Stock Preparation, Infection Experiment, and Virus Passage

Virus stock was made by transfection and infection of BHK-21 cells. Briefly, BHK-21 cells were seeded to 12-well plates 16–20 h prior to transfection and allowed to grow to 80% confluence for transfection. One microgram of D19044_7M plasmid (or D19044_4M; D19044_WT) and 0.5 μg of pTet-off plasmid were mixed and incubated in 200 μL of Opti-MEM medium containing 2.25 μL of ExFect transfection reagent (Vazyme, Nanjing, China) for 20 min. The plasmid mix was transfected to BHK-21 cells and cultured at 37 °C for 6 h, and then transfection medium was replaced with DMEM supplemented with 2% FBS and left for 2 days. The transfected culture was split every 2 days and supernatant was harvested at days 2, 4, 6, and 8, filtered (0.2 μm), and stored at −80 °C for later use. To expand the virus stock or perform infection experiment, transfection-derived supernatant virus was inoculated to naïve BHK-21 cells or other cell lines for 2 h, and then infection supernatant was replaced with DMEM with 2% FBS. The cultured supernatant was harvested at time points as required and stored at −80 °C.

To adapt the virus for efficient infection in culture, we continuously passaged the virus to naïve BHK-21 cells. Briefly, naïve BHK-21 cells were inoculated with transfection-derived virus supernatant in DMEM with 2% FBS for 72 h. Culture supernatant was collected, filtered (0.2 μm), and designated as first passage virus. The first passage virus was inoculated to naïve BHK-21 cells for 72 h to generate second passage virus. We consecutively passaged the virus until a fast virus spread and more intense fluorescence DENV-positive cells were detected through IFA in the culture. The passage-recovered viruses were stored at −80 °C and viruses from passages 21, 36, and 37 were subjected to RNA extraction using MagZol Reagent (Magen, Guangzhou, China). The cDNA was synthesized by reverse transcription using SuperScript III Reverse Transcriptase (Invitrogen, CA, USA) and amplified by four overlapping fragments (Section 2.2 and Table 1). The PCR products were analyzed by Sanger sequencing to identify if any substitution had occurred.

### 2.6. Virus Titration and Indirect Immunofluorescence Foci Assay (IFA)

The DENV titer was determined by indirect immunofluorescence foci assay (IFA) using BHK-21 cells [24,25] and expressed as focus forming unit per ml (FFU/mL). Briefly, BHK-21 cells were seeded in 96-well plates (4 × 10^3^ cells/well) and grown for 16–20 h. The virus supernatant was prepared as 10-fold serial dilutions in DMEM (from 10^0^ to 10^6^ dilutions), inoculated to BHK-21 cells, and left at 37 °C for 2 h. The cells were then washed once with DMEM, and incubated in DMEM containing 2% FBS for 48 h. Then, the culture supernatant was removed, and the cells were fixed using methanol (−20 °C) for 10–15 min. The fixed cells were incubated with DENV NS3 antibody (1:2000 dilution; GTX124252, GeneTex, CA, USA) at 4 °C overnight. To visualize DENV infected cells, the secondary antibody Alex Fluor 488 Goat Anti-Mouse IgG (H + L) (1:1000; Invitrogen, CA, USA) and cell nucleus staining dye Hoechst 33258 (1:1000; Invitrogen, CA, USA) were added at room temperature and incubated for 1 h. The numbers of FFU were manually counted using a fluorescence microscope (Leica Microsystems, DMI 4000B, Weizla, Germany).

### 2.7. Cytotoxicity Assay

BHK-21 cells were seeded in a 96-well plate (4 × 10^3^ cells/well) and incubated for 16–20 h. The cells were incubated with different concentrations of drugs for 48 h, and 10 μL of Cell Counting Kit-8 (CCK8, Dojindo, Kumamoto, Japan) was added to each well, gently shaken and incubated at 37 °C for 90 min. The optical density (OD_450_) was measured by a microplate reader (BioTek ELx800, Vermont, Germany).

### 2.8. Antiviral Drug Treatment

Mycophenolic acid (MPA, T1335, TargetMol, Shanghai, China) was dissolved to 10 mM using DMSO, aliquoted, and stored at −80 °C. Moxifloxacin hydrochloride (Tianjin Chasesun Pharmaceutical, Tianjin, China) was preserved at room temperature. Before treatment experiment, drugs were diluted to different concentrations (below cytotoxicity concentration) required for experiments using DMEM with 2% FBS. For antiviral treatment, BHK-21 cells were seeded in a 96-well plate (4 × 10^3^ cells/well) and cultured for 16–20 h. The cells were infected with virus dilution at multiplicity of infection (MOI) of 0.1 containing drug of different concentration for 2 h. The treatment medium was removed, and the cells were washed with DMEM and incubated in DMEM with 2% FBS containing drugs for 48 h. The cells were fixed and subjected to IFA, and the FFU were enumerated for each well.

## 3. Results

### 3.1. Genome Sequence of DENV-1 Genotype I Isolate, D19044

**Figure 1:**
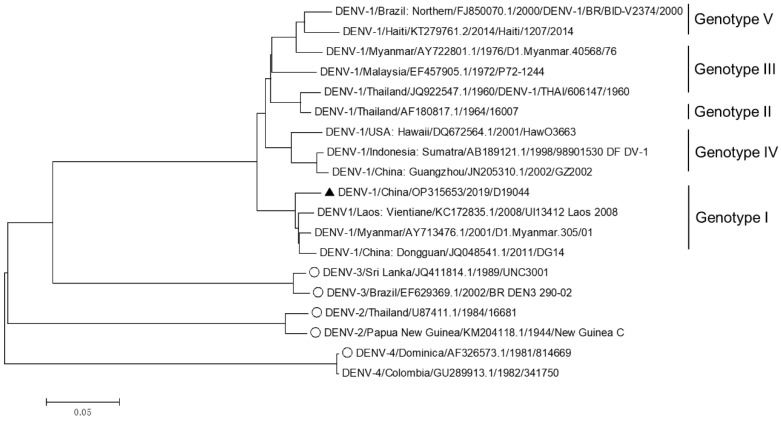
Phylogenetic analysis of D19044 and other DENV isolates. The isolates of genotypes I-V DENV-1 with complete genome sequences available from GenBank were selected to make phylogenetic analysis. Of DENV-2, -3, and -4, two sequences representative of each serotype were selected, especially for those with infectious clones developed. The phylogenetic tree is made based on the ORF nucleotide sequences using neighbor-joining method. A solid triangle (▲) highlights the D19044 isolate of this study. A circle (○) indicates that the infectious clone of the isolate has been developed.

DENV-1-infected serum D19044 was collected from a patient in Jieyang city, Guangdong province, China, in 2019. The patient serum was inoculated to C6/36 cells, and culture supernatant was collected at day 6 post infection. A part of C6/36 culture supernatant was inoculated to Vero cells and consecutively passaged up to 16 passages, followed by 13 passages in BHK-21 cells; unfortunately, the virus did not spread up in the cultures, without detectable viral titers. The other part of supernatant was subjected to RNA extraction and amplified by four overlapping DENV-1-specific RT-PCRs (Table 1). The PCR products were analyzed by Sanger sequencing, and the sequence covering genome nucleotides (nts) 50-10508 was obtained and designated D19044 wild-type (WT) sequence (OP315653). The D19044 WT sequence was phylogenetically analyzed against twelve sequences of DENV-1 genotypes I-V and eight sequences of DENV-2, -3, and -4 (two sequences for each) from GenBank (Figure 1). The results showed that D19044 was genetically close to the strain UI13412 (KC172835.1), which belongs to genotype I of DENV-1 (Figure 1). BLAST analysis revealed that D19044 was highly homologous to GZ8H/2019180/2019/I (MW261838.1), a clinical serum from Guangzhou in 2019, differing by 13 nucleotides and 2 amino acids (nt T1650C in E sequence with aa change I519T; G9452A in NS5, V3120I). Additionally, D19044 shared 97.8% and 99.49% identity at nucleotide and amino acids levels with infectious clone of genotype I isolate DENV1-3 C_0268-96 (OK605755.1), respectively [17]. Thus, D19044 was a genotype I isolate of DENV-1 recently prevalent in south China.

### 3.2. Construction of Full-Length D19044 cDNA Clone

**Figure 2:**
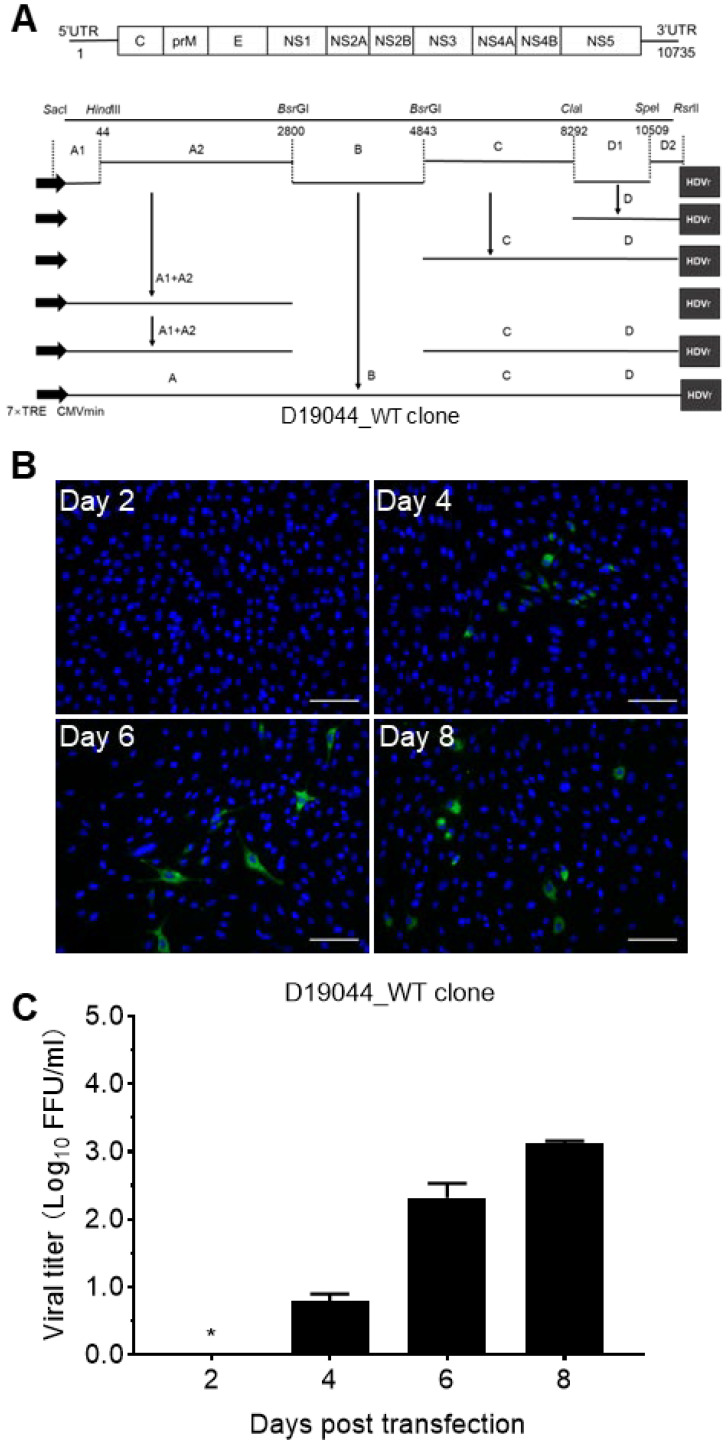
Construction of D19044 clone and its capacity to generate infectious virus particles. (**A**) Schematic diagram of construction of full-length D19044 cDNA clone. The full-length genome was illustrated and divided into four overlapping subgenomic fragments (A–D) for the convenience of cloning, of which fragment (A) was subdivided into A1 and A2, and (D) was subdivided into D1 and D2. The key restriction sites and nucleotide positions are indicated for fragment A (nts 1-2805, *Sac*I*-Bsr*GI), B (nts 2724-4897, *Bsr*GI*-Bsr*GI), C (nts 4755-8347, *Bsr*GI*-Cla*I) and D (nts 7485-10735, *Cla*I*-Rsr*II). The pTight vector contains 7 × TRE and CMVmin, as well as hepatitis D virus ribosome (HDVr). The order of cloning fragments was as the followings: B → A → C → D (see Results), as indicated by solid lines in the constructs. Finally, a full-length D19044 wild-type (D19044_WT) clone was made. (**B**) D19044_WT clone released infectious virus particles from BHK-21 cells. The D19044_WT clone plasmid was transfected into BHK-21 cells, and DENV-1 positive cells were monitored by immunofluorescence assay (IFA) for DENV NS3 protein (green) and cell nuclei (blue). The images were taken from one transfection experiment. Bar, scale 100 μm. (**C**) The viral titer of D19044_WT after transfection of BHK-21 cells. The culture supernatant collected at different time points of post transfection (p.t.) and subjected to FFU assay. The mean ± standard error of the mean (SEM) from three independent experiments are shown. An asterisk (*) indicates the viral titer was below the limit of detection (LOD; < 0.69 log_10_FFU/mL).

To overcome the instability of DENV cDNA clone in transformant *E. coli* bacteria, we selected a low-copy plasmid pTight, which was modified from pBR322 [19,20]. We and others have previously used this vector for development of infectious clones of JEV, DENV, and ZIKV [18,19]. To construct a D19044 full-length clone, we divided its genomic cDNA into four fragments A, B, C, and D, by restriction sites (Figure 2A); fragment A was fused from sub-fragments A1 and A2, while D was a fusion of D1 and D2 (Figure 2A and Section 2). Partial sequences of 5′UTR (nts 1–49, mainly A1 fragment) and 3′UTR (nts 10,509–10,735, D2 fragment) were obtained from isolate GZ2002 (JN205310) [21]. Inexplicably, we found that the cloning was restricted and selective by the order of fragment cloning into the pTight vector. When fragments were cloned in the order of C → D → A, namely fragment A was first cloned into the vector to get pTight-A, followed by adding D to pTight_A to make pTight_A-D, then C was added in to get pTight_A-C-D, all 70 colonies from three independent ligation-transformation experiments carried multiple nucleotides and/or amino acid substitutions or deletions. In subsequent attempts, we tested different orders of fragment cloning and found that D19044 full-length clone could be constructed only in the order B → A → C → D, namely D was first cloned to get pTight_D, followed by adding C to make pTight_C-D, and then adding A to obtain pTight_A-C-D. Finally, B was added to generate full-length D19044 wild-type cDNA clone (D19044_WT). All subclones and final D19044_WT clone were confirmed by Sanger sequencing (Figure 2A).

### 3.3. Transfection of D19044 Clone Plasmid Produced Infectious DENV Particles

To test the capacity of producing infectious particles for D19044_WT clone, we transfected D19044_WT plasmid together with pTet-off plasmid into five different types of cells, A549, 293T, Huh7.5.1, Vero, and BHK-21 cells. The plasmid of pTet-off expresses the tet-responsive transcriptional activator (tTA), which will bind to a tet-responsive element (TRE) in the pTight plasmid to activate the CMVmin promoter-controlled transcription to produce viral genomic RNA, thus initiating translation and replication of D19044_WT RNA [18,19]. The results showed that only the BHK-21 culture showed a greater number of DENV positive cells with intense fluorescence by the IFA method (Figure 2B), and other cell cultures showed weak or undistinguishable fluorescence signals. Thus, we selected BHK-21 cells for subsequent experiments, without further optimizing the transfection for other cell lines. Noting that, we did not observe apparent cytopathic effect or cell death in infected culture and did not succeed for plaque forming assay, as described previously [18], thus we monitored D19044 virus infection by NS3-specific immunofluorescence method in this study. The culture supernatant was collected at days 2, 4, 6, and 8 post transfection (p.t.), and viral titers were determined by inoculating supernatant to naïve BHK-21 cells for FFU assay. The results showed that D19044_WT transfection of BHK-21 cells released infectious virus, however, the viral titer was low (1.26 × 10^3^ FFU/mL; day 8 p.t.) (Figure 2C).

To further adapt the D19044_WT virus, we continuously passaged the virus to naïve BHK-21 cells, determined the viral titer regularly, and found that the viral titer rose slightly at passage 13 (47 days from transfection; ~8 × 10^3^ FFU/mL), claiming a further adaptation. When reaching passage 34, the viral titer rose significantly (3.9 × 10^5^ FFU/mL), thus we expanded this virus stock in the following passages and terminated at passage 37. To identify adaptive acid substitution that may have acquired for the enhanced viral titer, we sequenced D19044 ORF region for passages 21, 36, and 37 by Sanger sequencing. Seven amino acid substitutions (7M) were identified, including G2084A (aa change G664K in E), C4410T (T1439M in NS2B), and G6143A (D2017N in NS3) in the 21th passage virus, T7161C (V2356A in NS4B) and C7689T (S2532F in NS5) in the 36th passage virus, and A1125C (K344T in E) and C1416T (T441I in E) in the 37th passage virus (Table 2).

### 3.4. Efficient Virus Production of D19044 Clone Carrying Amino Acid Substitutions

**Figure 3:**
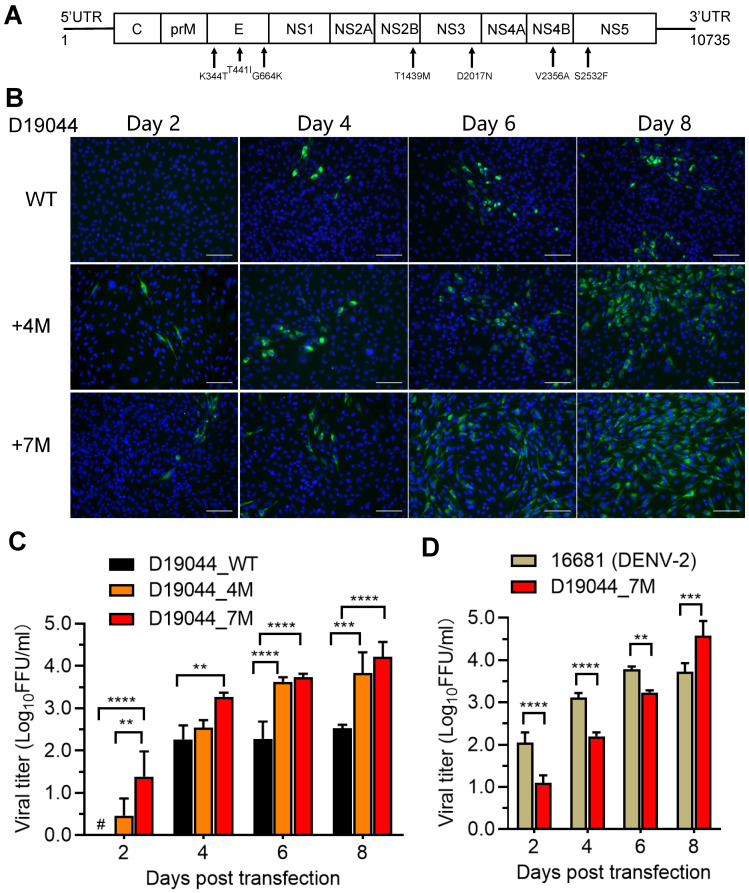
Amino acid substitutions improved the virus production of D19044 clone. (**A**) Diagram of DENV-1 genome with seven amino acid substitutions (7M) identified from passage-recovered viruses. (**B**) IFA of D19044_WT, D19044_4M, and D19044_7M after plasmid transfection of BHK-21 cells. DENV-1 NS3 protein (green) and cell nuclei (blue) were visualized. Bar, scale 100 μm. (**C**) The DENV titer of culture supernatant. Three D19044 clones (D19044_WT, _4M, and _7M) were transfected into BHK-21 cells, and the supernatant were collected for FFU assay at time points as indicated. The means ± SEM of three independent experiments are shown. A “#” indicates the titer was below LOD (<0.69 log_10_FFU/mL). (**D**) Comparison of viral titers released by D19044_7M and DENV-2 clone 16681. BHK-21 cells were transfected with D19044_7M and DENV-2 16681 plasmids, and the supernatant were collected for FFU assay at time points as indicated. The means ± SEM of three independent experiments are shown. In panels **C** and **D**, statistics analyses were performed using multiple comparison test in a two-way ANOVA. ** *p* < 0.01; *** *p* < 0.001; **** *p* < 0.0001.

To test the adaptation effect of passage-derived amino acid substitutions, we engineered them back into a D19044_WT clone. Since V2356A was partially changed in passage 21 (1 × 10^3^ FFU/mL) and became completely changed in passage 36, we initiated to test the effect of four amino acid substitutions (4M, G664K/T1439M/D2017N/V2356A) and 7M in D19044_WT and designated D19044_4M and D19044_7M, respectively (Figure 3A). Both plasmids were transformed into competent Turbo *E. coli* bacteria and transformant colonies appeared after 24 h incubation. D19044_7M acquired G3384T (aa R1097L in NS1), which may stabilize its sequence in bacteria, and we kept this change in D19044_7M clone.

To determine whether amino acid substitutions enhance virus production, we transfected D19044_4M, D19044_7M, and D19044_WT clones into BHK-21 cells and examined the viral titers of culture supernatant at different time points post transfection (p.t.) (Figure 3B,C). D19044_4M and D19044_7M produced DENV, with detectable titers at day 2 p.t., while D19044_WT was undetectable. At day 4 p.t., virus spread and titers of D19044_7M were higher than that of D19044_4M and D19044_WT by 5.1~8-fold; at days 6 and 8, D19044_4M and D19044_7M were higher than D19044_WT by 16.5~20.7 and 29.8~60.1 fold, respectively (Figure 3C). D19044_7M reached peak titer (7.5 × 10^4^ FFU/mL) at day 8 p.t. and higher than D19044_4M (2.24 × 10^4^ FFU/mL) (Figure 3C). The recovered viruses were confirmed by sequencing analysis, and all amino acid substitutions engineered were maintained. Thus, passage-recovered amino acid substitutions promoted virus production of D19044 clone, and D19044_7M represented most efficient clone for subsequent experiments.

Since DENV-2 clones were used in the field more frequently, we compared virus production of D19044_7M to a DENV-2 infectious clone, 16681, by plasmid transfection of BHK-21 cells [19,26,27]. The results showed that D19044_7M produced lower viral titers than 16681 clone at days 2, 4, and 6, but surpassed 16681 at day 8 (Figure 3D). Noting that, 16681 tended to cause cell death from day 6 p.t., whereas D19044_7M did not apparently cause cell death, which may contribute to a higher titer for D19044_7M at day 8 (Figure 3D).

### 3.5. D19044_7M Clone Was Genetically Stable in Bacteria after Multiple Transformation-Purification Cycles

It is an unsolicited issue that flavivirus cDNA encounters instability in transformant recipient bacteria [20,28,29], thus we proceed to test the bacterial stability of D19044_7M clone by repeating transformation-purification cycles (TPC) as previous described [18]. Briefly, D19044_7M plasmid was transformed into Turbo competent bacteria, single colonies were picked and cultured in 2 × YT broth, and plasmid DNA was extracted and transformed into competent Turbo bacteria again. We repeated five rounds of the transformation-purification procedure and confirmed the sequence of plasmid. One amino acid substitution was identified (NS4B, nt C7158G, aa A2355G) in the recovered plasmid and designated D19044_7M/5TPC.

**Figure 4:**
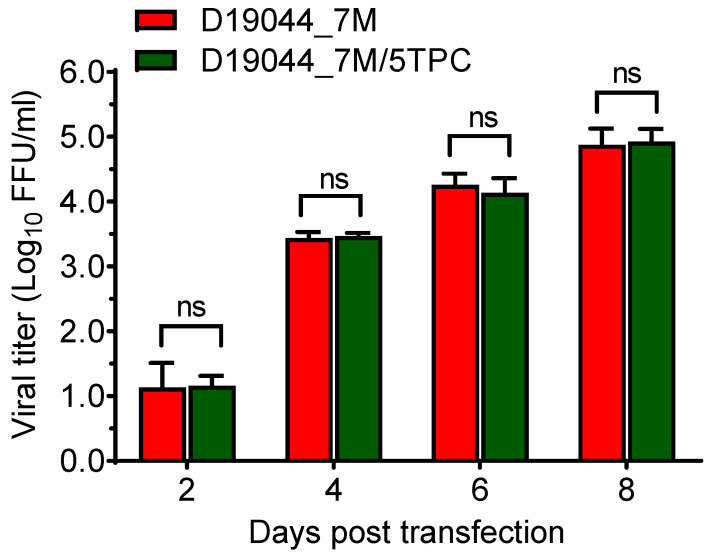
D19044_7M clone were genetically stable in the transformant bacteria. D19044_7M clone plasmid that have gone through five rounds of transformation-purification cycle (5TPC) produced infectious virus comparable to D19044_7M clone. The viral titers were determined by FFU assay. The means ± SEM of three independent experiments are shown. Statistics analysis were performed using multiple comparison test in a two-way ANOVA. ns, no significance.

Next, we tested the capacity of producing viral particles for D19044_7M/5TPC in comparison to D19044_7M. Both clones released infectious viruses with comparable viral titers at days 2, 4, 6, and 8 following plasmid transfection of BHK-21 cells. Peak viral titers peaked at day 8 (1 × 10^5^ FFU/mL), indicating that A2355G did not affect production of infectious virus (Figure 4). Moreover, no other deletion and amino acid substitution was found in the genome, especially in substitution- and deletion-prone region, prM-E-NS1 [18,30]. Thus, we considered D19044_7M recombinant was genetically stable in bacteria, feasible for molecular manipulation.

### 3.6. Differential Efficiency of D19044_7M and D19044_WT in Various Vell Lines

**Figure 5:**
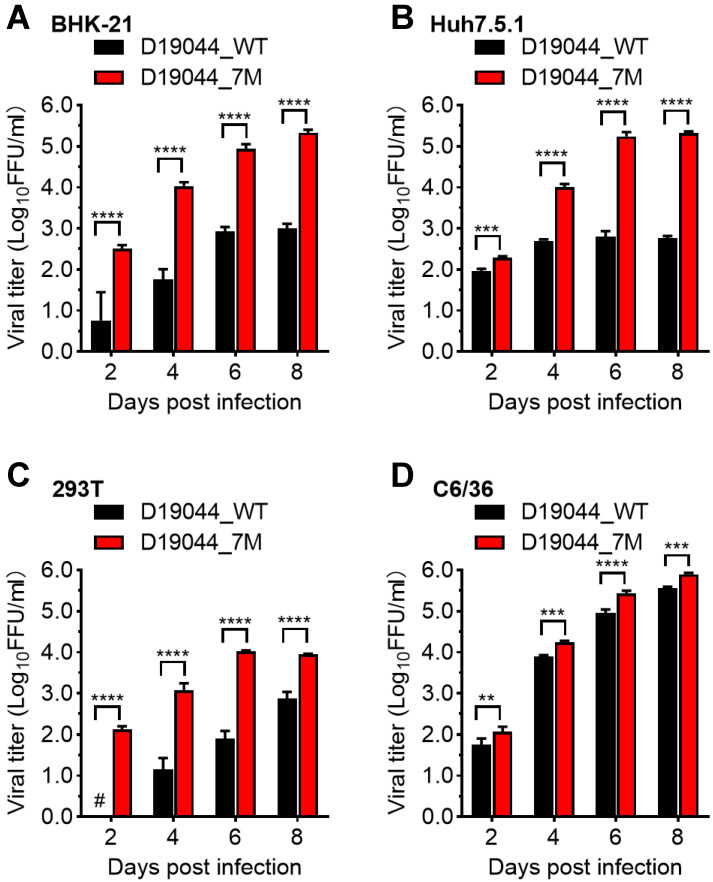
Virus production of D19044_WT and D19044_7M in various cell lines. BHK-21 cells (**A**), Huh7.5.1 cells (**B**), 293T cells (**C**), and C6/36 cells (**D**) were infected with D19044_WT and D19044_7M (MOI = 0.001). The viral titers released in supernatant were determined by FFU assay. A “#” indicates the titer was below LOD (<0.69 log_10_FFU/mL). The means ± SEM of three independent experiments are shown. Statistics analysis were performed using multiple comparison test in a two-way ANOVA. ** *p* < 0.01; *** *p* < 0.001; **** *p* < 0.0001.

Since D19044_7M was generated by adapting D19044_WT in BHK-21 cells through serial passages and it carried seven amino acid substitutions, we wanted to test whether it was capable of infecting other cell lines. We infected BHK-21, Huh7.5.1, 293T, and C6/36 cells with same MOI of viruses (MOI = 0.01; ~70% culture confluence) and determined the viral titers released at days 2, 4, 6, and 8 p.i. (Figure 5). The results showed that all cell lines were infected by both viruses, however D19044_7M was more efficient in BHK-21, Huh7.5.1, and C6/36 cells (peak titers, 1 × 10^5.3^–10^5.9^ FFU/mL) than in 293T cells (1 × 10^4.0^ FFU/mL) (Figure 5A–D). D19044_WT was apparently less efficient than D19044_7M by 75~268-fold in BHK-21, Huh7.5.1, and 293T cells at days 4 and 6, however similar to D19044_7M in C6/36 cells (~2-folds) (Figure 5A–D). Together, D19044_7M was more adapted for infection of vertebrate cell lines, without loss infectivity to the mosquito cell line.

### 3.7. D19044_7M Virus Was Inhibited by Antiviral Compound

**Figure 6:**
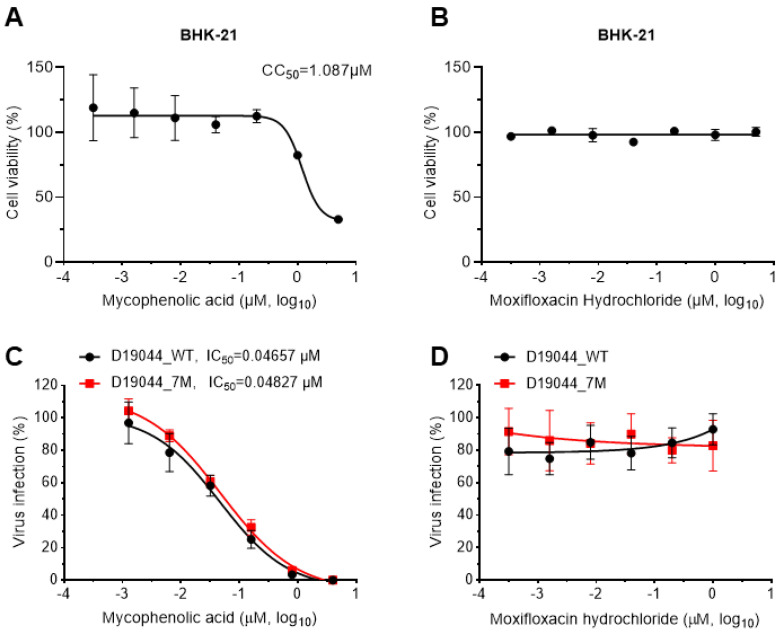
D19044 was inhibited antiviral compound in a dose-dependent manner. (**A**,**B**) The cell viability of BHK-21 cells under the treatment of mycophenolic acid (MPA) and moxifloxacin hydrochloride. (**C**) MPA inhibited D19044_7M and D19044_WT in a dose-dependent manner with comparable IC_50_ (0.04827 μM and 0.04657 μM). (**D**) Moxifloxacin hydrochloride did not inhibit D19044_7M and D19044_WT viruses. In **C** and **D**, BHK-21 cells were infected with D19044_7M and D19044_WT with MOI of 0.1 for 2 h, and then incubated with drugs of different concentrations for 48 h. The infection rates were examined by IFA and normalized to the nontreated mock controls. The means ± SEM of four determinations are shown.

An infectious clone provides a valuable tool for the study of various aspects of DENV virology, of which one most expected may be its feasibility for testing antivirals. We proceeded to test its responsiveness against mycophenolic acid (MPA). MPA inhibits DENV infection by preventing accumulation of positive- and negative-strand RNAs [2] and has been shown to suppress ZIKV infection in different cell types [31,32]. Moxifloxacin hydrochloride was selected as the negative control, which is used for treatment of pulmonary infections and has no inhibition to flaviviruses [33]. The antiviral treatment was performed below the cytotoxic concentration of the drugs (BHK-21 cells, MPA CC_50_ = 1.087 μM) (Figure 6A,B). BHK-21 cells in 96-well plates were infected with D19044_WT and D19044_7M (MOI = 0.1) and then treated with drugs of serial dilution. The results showed that D19044_7M (IC_50_ = 0.04827 μM) and D19044_WT (IC_50_ = 0.04657 μM) were inhibited by MPA in a dose-dependent manner; the IC_50_ comparable for both viruses indicate that amino acid substitutions (7M) did not affect viral response to MPA treatment (Figure 6C). Moxifloxacin hydrochloride did not inhibit virus infection (Figure 6D). Together, these data demonstrate that D19044_7M could serve as a tool for the study of anti-DENV drugs.

## 4. Discussion

In this study, we constructed a DNA-launched full-length infectious clone for a DENV-1 genotype I isolate, D19044. Through serial passages in cell culture, seven amino acid substitutions (7M) were identified and subsequently demonstrated to promote efficient virus production in vitro. The final D19044_7M clone was able to release infectious viruses with peak titers of 7.5 × 10^4^ FFU/mL and was genetically stable in transformant bacteria, thus enabling its usefulness to the field. Both D19044_WT and D19044_7M virus showed infections to BHK-21, 293T, Huh7.5.1, and C6/36 cells, and were inhibited by MPA drug in a dose-dependent manner. The development of a full-length infectious cDNA clone for a clinical isolate provides a useful tool for the study of DENV, especially for the serotype I DENV-1.

There are different ways to develop infectious clones for DENV isolates, and vector selection plays a key role for assembling a full-length cDNA clone with genetic stability in transformant bacteria. Different plasmid vectors have been used, including high copy number plasmid vector for a DENV-2 isolate [26], low copy number plasmid vectors for DENV-1 [34], DENV-2 [27,35], and DENV-4 [36], bacterial artificial chromosomes for DENV-1 [37] and DENV-2 [38]. Yeast artificial chromosomes [39] and shuttle vector [40,41,42] have also been used. Recently, 20 infectious clones covering DENV-1-4 were constructed using the circular polymerase extension reaction method [17,43], thus wisely bypassing propagation of flavivirus cDNA in bacteria and yeast, which was previously used for developing infectious clones of flaviviruses. In addition to vector selection, other strategies to stabilize the cDNA clones include inserting intron sequences to interrupt the putative *E. coli* promoters (ECP) [28,44] or introducing silent substitutions into the putative ECP sequence [18,20]. Moreover, methods avoiding transformation step have also been applied, such as long PCR [45], infectious-subgenomic-amplicons [46], and subgenomic plasmid recombination method (SuPReMe) [47].

We selected pTight vector to construct the D19044 full-length clone, as this vector is a low copy number plasmid originally modified from pBR322 [19], containing 7 × TRE and CMVmin promoter upstream of the cloned viral cDNA sequence. The 7 × TRE could inhibit the potential ECP activity that a viral cDNA sequence may have, thus permitting plasmid replication in bacteria and making laboratory manipulation feasible [18,19]. The CMVmin promoter-controlled vector allows a DNA-launched production of viral genomic RNA transcripts by plasmid transfection of eukaryotic cells, thus avoiding in vitro transcription and RNA manipulation. In addition, the CMVmin promoter vector also showed advantages in maintaining the stability of flavivirus genome in transformant bacteria, such as C41 and Turbo *E. coli* bacteria [18,19]. Nevertheless, although these elements noticeably reduced the ECP activity, we identified deletions, neucleotide and/or amino acid substitutions, or recombination in transformation-recovered plasmids, mainly in prM-E-NS1 region, when we ligated four fragments (A, B, C, and D) in the order of C → D → A. Thus, we tried different orders of assembling and only succeeded when these fragments were cloned in the order of B → A → C → D (Figure 2A). Noted that, colonies with mutagenesis events generally showed abnormal manifestation, such as a faster growth and a larger colony size; mutagenesis events may have eliminated or reduced toxic proteins producing in bacteria, thus bacteria gained a growth benefit. In contrast, bacteria colonies carrying correct viral cDNA usually grew slower, became identifiable after 24 h incubation, and had a smaller size. Through the use of the pTight vector and a specific order of fragment cloning, we generated a D19044_7M clone with genetic stability in the transformant bacteria. A full-length clone genetically stable in bacteria is vitally important and allows its utility for molecular manipulation.

Adaptive amino acid substitutions are often required to improve virus production of infectious clones [18,48,49]. D19044_WT clone showed a slow virus spread and low-level infectious virus after transfection (Figure 2 and Figure 3), but became efficient after a number of virus passages (e.g., up to 34-37 passages, 3.9 × 10^5^ FFU/mL). Seven amino acid substitutions (7M) were identified (Table 2) and proven to greatly improve virus production (1 × 10^5^ FFU/mL). Therefore, the acquisition of adaptive amino acid substitutions by serial viral passage in the culture is a strategy recommendable for making an efficient infectious clone for DENV and other RNA viruses. Here, it is interesting that addition of 7M improved the virus infection greatly in the cell lines from human and Hamster Syrian, but to a less extent in mosquito C6/36 cells (Figure 5D). D19044_WT virus infected mosquito C6/36 cells more efficiently than other cell lines, suggesting that this virus may be more adaptive to C6/36 cells or mosquitos’ phycological environment than in other species. These results may be due to the fact that the D19044_WT virus was generated from the cDNA clone constructed from infected-C6/36 cells after 6 days. It is unknown whether there was adaptive substitution(s) had been introduced into the viral genome for an efficient infection of C6/36 cells (without patient serum adequate for sequencing). However, in this case, 7M had proven a potential in culture adaptation of cross-species, thus the functional roles of 7M in the viral life cycle and cross-species adaptation in vitro and in vivo warrant future investigations.

The wild-type virus used in this study was generated from D19044_WT cDNA clone that was made from infection supernatant of C3/36 cells (day 6), due to a limited volume of patient serum available. The C6/36-deirived supernatant showed very low infection in Vero and BHK-21 cells after a number of passages without detectable viral titers, thus we were unable to compare the infection of D19044_7M and original serum virus in cultured cells. In addition, the functional roles of 7M and potential effect of partial 5′UTR and 3′UTR sequences from GZ2002 used in D19044_7M clone were not explored. These limitations of this study warrant future investigations towards an in-depth characterization and utility of D19044_7M clone.

In summary, we have developed an efficient infectious clone for a genotype I DENV-1 isolate that was recently prevalent in south China. Given that genotype I virus is predominantly circulating in different regions and is responsible for several major outbreaks, D19044_7M will be highly relevant for the study of DENV-1.

## Figures and Tables

**Table 1:** Primers were used for RT-PCR of DENV-1 isolate, D19044.

Primers	Position	Sequence (5′-3′)	Product Length (nt)
F17	17–37	GGACCGACAAGAACAGTTTCG	3331
R3347	3324–3347	CATGAATTATCTTCCCTGTGACTG	
F2724	2724–2743	AAATGATTAGGCCACAACCC	2174
R4897	4877–4897	AATGGCTCCAACTTCACCTTC	
F4755	4755–4778	ATGGAGGAGGTTGGAGGCTTCAAG	3593
R8347	8324–8347	GCCTAGGTCCACGTCTCTTTCATA	
F7485	7485–7510	TTTCCATGGCAAACATTTTCAGAGGA	3060
R10544	10,524–10,544	GTTGTGTCTTGGGAGGGGTCT	
F-5′UTR	Vector-15	gagctcgtttagtgaaccgtcAGTTGTTAGTCTACG	70
R-5′UTR	15–49	AAGCTTCCGATTTGAAACTGTTCTTGTCGGTCCAC	
F-3′UTR	10,509–10,529	ACTAGTGGTTAGAGGAGACCC	277
R-3′UTR	Vector	ttcggatgcccaggtcggaccgcgaggaggtggagat	
R6260 *	6239–6260	CCACGTCCATGTTCTCCTCCAA	
R10654 *	10,633–10,654	GGTCTCTCCCAGCGTCAATATG	

*, the primers were used for reverse transcription. Lowercases indicate the sequences priming to the vector sequence.

**Table 2:** Amino acid substitutions identified in the recovered viruses after serial passages.

Number of Passage	Nucleotide Position	Nucleotide Change *	Gene Region	Amino Acid Position	Amino Acid Change
21	2084	G–A	E	664	Glu–Lys
21	4410	C–T	NS2B	1439	Thr–Met
21	6143	G–A	NS3	2017	Asp–Asn
36	7161	T–C/t	NS4B	2356	Val–Ala
36	7689	C–C/t	NS5	2532	Ser–Phe
37	1125	A–C	E	344	Lys–Thr
37	1416	C–T/c	E	441	Thr–Ile

*, Capital letters indicate complete change (or >50% change) in sequencing reads; lowercases indicate a minor change (<50%) in sequencing reads.

## Data Availability

All experimental data are present in the manuscript. Sequence data has been deposited in GenBank. Plasmids are available upon request.
